# Changes in Compression Pressure of Elastic Stockings for the Lower Limbs During Cesarean Section: A Prospective Observational Study

**DOI:** 10.7759/cureus.62809

**Published:** 2024-06-21

**Authors:** Shunsuke Hyuga, Hiroaki Kondo, Tomoe Fujita, Toshiyuki Okutomi

**Affiliations:** 1 Division of Obstetric Anesthesia, Center for Perinatal Care, Child Health and Development, Kitasato University Hospital, Sagamihara, JPN

**Keywords:** entrapment neuropathy, peripheral nerve injuries, thromboembolism, postpartum, neuropathy, elastic stockings, cesarean section

## Abstract

Background

Postpartum peripheral nerve injuries can impact recovery. Elastic stockings are recommended for thromboembolism prevention, although concerns about entrapment neuropathy exist. In this prospective observational study, we investigated the differential compressions caused by wearing elastic stockings before and after anesthesia, as well as changes in the diameters of the lower leg and ankle in parturient women undergoing spinal anesthesia for elective cesarean section (CS).

Methods

Eighteen pregnant women, classified by the American Society of Anesthesiologists as having physical status 2, underwent lower leg measurements taken before a CS. Elastic stockings were applied, and compression pressure was measured at pre-anesthesia, post-surgery, and six hours post-return to a hospital room. Fluid, blood loss, urine output, and neuropathy presence were recorded. For all parameters, changes at the three time points were compared for the primary analysis. For secondary analysis, participants were categorized as having intraoperative blood loss greater than (group P) or less than 1,000 g (group N), and factors were compared with pre-anesthesia and six hours post-return to a room. Data were analyzed and presented using a one-way analysis of variance with Bonferroni correction for multiple comparisons or unpaired two-tailed t-tests for pairwise comparison.

Results

None of the women had postoperative entrapment neuropathy. Six patients had >1,000 g of blood loss. Compression significantly increased from pre-anesthesia (left 13.6 ± 2.4, 95% CI: 12.18 to 14.52; right 13.4 ± 2.4, 95% CI: 12.41 to 14.69) to post-surgery (left, 17.4 ± 2.6, 95% CI: 15.68 to 18.12; right, 16.9 ± 2.6, 95% CI: 16.20 to 18.70) (p < 0.01). Compression pressure at post-surgery differed significantly between group P (left, 15.3 ± 1.3; right, 14.7 ± 1.8; 95% CI: -4.98 to -0.32) and group N (left, 18.1 ± 2.9; right, 17.8 ± 2.4; 95% CI: -5.38 to -0.26) (p < 0.05). The results are expressed as mean ± standard deviation, with P-values <0.05 indicating statistical significance.

Conclusions

In this study, no neuropathy occurred; however, over-compression risk with elastic stockings, especially when exceeding recommended pressure levels, was highlighted. Balancing thromboembolism prevention and over-compression risks is crucial for patients undergoing CSs with spinal anesthesia.

## Introduction

The prevalence of postpartum peripheral nerve injuries occurs in approximately 0.3-2% of all deliveries [1], which outnumbers neurological insults directly associated with neuraxial anesthesia [2]. Intrinsic obstetric palsies from either compression or nerve stretch during delivery are a major cause of these nerve injuries; peripheral nerve injury can interfere not only with patient postoperative recovery but also with child rearing, which may contribute to patient anxiety, leading to postpartum depression [1]. Nevertheless, prophylactic measures, such as wearing elastic stockings, intermittent pneumatic compression, and anticoagulant therapies, are needed to reduce the risks of perioperative pulmonary thromboembolism; these measures are recommended in the Enhanced Recovery After Cesarean guidelines aimed at enhancing recovery after cesarean section (CS) surgery [3]. However, overzealous compression of the lower limbs by elastic stockings may cause entrapment neuropathy [4]. Moreover, spinal or epidural anesthesia for a CS inevitably induces postoperative sensory deficits in the lower extremities, thereby masking symptoms of nerve occlusion. In addition, local anesthetic sensory blockade is a risk factor for peripheral neuropathy, as it delays the sensory perception of neuropathy [5]. Despite this potential risk of postoperative peripheral neuropathy, no study has probed the impact of compression pressure on the lower limbs with elastic stockings during a CS under spinal anesthesia. Herein, we evaluated the differential compressions induced by the wear of elastic stockings between pre- and post-anesthesia, as well as alterations in diameters of the lower leg and ankle in parturient women who received spinal anesthesia during CS.

## Materials and methods

In adherence with the Strengthening the Reporting of Observational Studies in Epidemiology guidelines, we conducted a prospective observational study at Kitasato University Hospital, Sagamihara, Japan, ratified by our institutional review board. All participants were fully informed and provided written informed consent. Ethical approval for this study was provided by the Kitasato University Medical Ethics Organization on January 4, 2019 (approval number B18-183). The clinical trial number and registration URL for this study are as follows: umin000035319 (https://center6.umin.ac.jp/cgi-bin/ctr/ctr_view_reg.cgi?recptno=R000040233).

From January 2019 to December 2021, we included 18 pregnant women (American Society of Anesthesiologists physical status 2) who underwent elective CS with spinal anesthesia. Participants with contraindications for spinal anesthesia, chronic medication usage, smoking habits, hypertension, diabetes, thyroid disease, or neuropathic complications were excluded. All participants wore medical elastic stockings that were commercially available in Japan (Toray Industries, Inc., Tokyo, Japan). One day before surgery, as recommended by the manufacturer, the diameter of the lower leg was measured at the thickest part of the calf and that of the ankle was measured at the thinnest part. Subsequently, appropriately sized elastic stockings were applied based on these measurements. For the primary analysis, compression pressure with the stockings was measured on each of the left and right legs at three distinct time points (pre-anesthesia (PRE), post-surgery (POST), and six hours post-return to the hospital room (6HR)) using the Kikuhime™ pressure monitor (TT MediTrade, Sorø, Denmark) at the point where the lower leg diameter was measured. Ankle and lower leg diameters were also measured at the same points. PRE was defined as immediately before spinal anesthesia after entering the operating room, POST as immediately after the end of surgery, and 6HR as six hours after spinal anesthesia, respectively, and PRE was used as a control for comparison. Concurrently, the final level of anesthesia, intraoperative infusion and blood loss volumes, urine output, and presence or absence of neuropathy were also recorded. For the secondary analysis, the patients were divided into two groups: those with intraoperative blood loss greater than 1,000 g (group P) and those with intraoperative blood loss less than 1,000 g (group N), and ankle and lower leg diameters and compression pressures at the end of the surgery and six hours post-surgery were compared in the two groups. Patients with any missing data were not included in the analysis, but there were no missing data in all 18 patients. Spinal anesthesia for CS was performed with a 27-g pencil-point needle from the L3/4 intervertebral space and 12 mg of hyperbaric bupivacaine, 10 μg of fentanyl, and 100 μg of morphine hydrochloride. Extracellular fluid was started at 1,000 ml/hr as soon as the patient entered the operating room, and continuous intravenous phenylephrine at 1 mg/h was started as soon as induction of anesthesia was performed. When the systolic blood pressure was less than 80% of the blood pressure at rest in the ward, phenylephrine 0.05 mg was administered intravenously if the heart rate was greater than 70 beats per minute, or ephedrine 5 mg was administered if the heart rate was less than 70 beats per minute. Surgery was initiated after confirming that the cold-numbing zone had expanded to Th4-S5. After delivery, continuous intravenous oxytocin 2.5 IU/h was started, and oxytocin 1 IU or methylergometrine 0.2 mg was administered intravenously as needed according to the degree of uterine contractions. No sedatives of any kind were used during surgery.

Data were analyzed and presented using a one-way analysis of variance with Bonferroni correction for multiple comparisons or unpaired two-tailed t-tests for pairwise comparison, using GraphPad Prism (GraphPad Software, Boston, United States). The results are expressed as mean ± standard deviation, with P-values <0.05 indicating statistical significance. The minimum sample size, calculated a priori, was 18, with an effect size of 1.03, a type-I error of 0.05, and a power of 0.95, using G*Power software (Version 3.1.9.6; Heinrich-Heine-Universität Düsseldorf, Düsseldorf, Germany).

## Results

**Figure 1:**
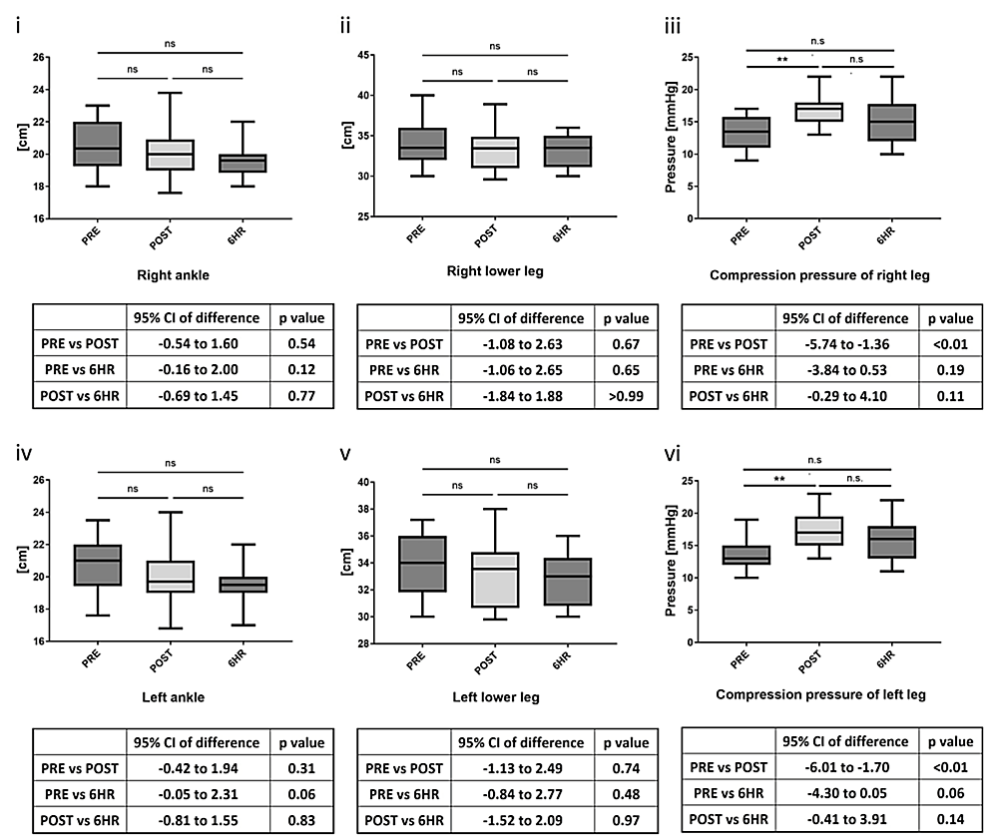
Circumferences of the ankles and lower legs and elastic stocking pressure at each point: (i) circumferences of the right ankle joints; (ii) circumferences of the right lower leg; (iii) elastic stocking pressure (right); (iv) circumferences of the left ankle joints; (v) circumferences of the left lower leg; and (vi) elastic stocking pressure (left) ** P < 0.01 6HR, six hours after anesthesia; ns, not significant; PRE, before anesthesia induction; POST, at the end of the surgery

**Figure 2:**
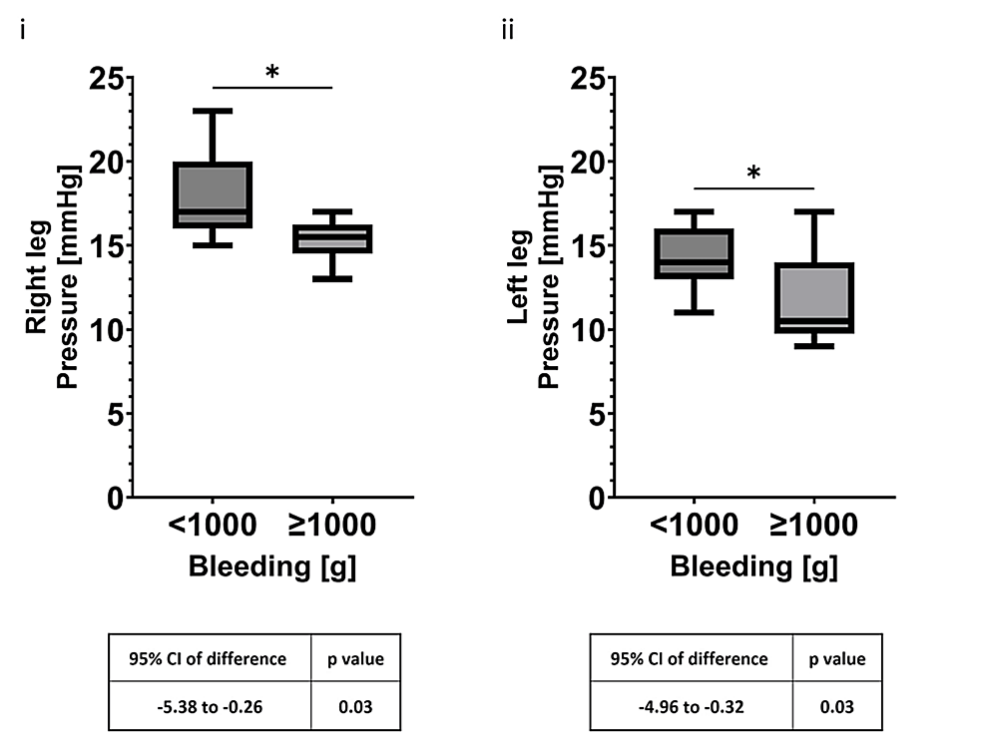
Intraoperative blood loss of more than 1,000 g (group P) compared with less than 1,000 g (group N): (i) comparison of compression pressure in the right lower leg between the two groups and (ii) comparison of compression pressure in the left lower leg between the two groups * P < 0.05 6HR, six hours after anesthesia; ns, not significant; POST, at the end of the surgery

No postoperative atonic hemorrhage or neuropathy was observed. Table 1 presents the patient demographics, final anesthesia level, intraoperative infusion volume, blood loss volume, and urine output. Intraoperative blood loss was greater than 1,000 g in six patients. Despite insignificant variances in the lower leg and ankle diameters in the PRE, POST, and 6HR measurements, compression significantly increased between the PRE (left, 13.6 ± 2.4 mmHg; right, 13.4 ± 2.4 mmHg) and POST (left, 17.4 ± 2.6 mmHg; right, 16.9 ± 2.6 mmHg; P < 0.01) measurements (Figure 1). In a secondary analysis comparing intraoperative blood loss of more or less than 1,000 g, there were no significant differences in ankle and lower leg diameters or in compression pressure at 6HR between the two groups. However, there was a significant difference (P < 0.05) in compression pressure at POST between group P (left, 15.3 ± 1.3 mmHg; right, 14.7 ± 1.8 mmHg) and group N (left, 18.1 ± 2.9 mmHg; right, 17.8 ± 2.4 mmHg) (Figure 2).

**Table 1:** Participant characteristics Values are presented as the mean ± standard deviation, n (%), or median (range) (n = 18).

Parameter	Value
Maternal age (years)	33.2 ± 3.2
Gestational age (weeks)	38.3 ± 1.0
Primipara (%)	11 (55)
Height (cm)	160.0 ± 4.0
Weight (kg)	64.3 ± 6.9
Non-gestational weight (kg)	55.2 ± 6.2
Bleeding (g)	911.9 ± 316.0
Infusion volume (mL)	1,196.0 ± 209.3
Urine (mL)	154.3 ± 96.6

## Discussion

Our investigation pioneers the assessment of temporal variations in compression exerted by elastic stockings during CS under spinal anesthesia. Remarkably, despite unchanged ankle joint and lower leg circumferences, we observed a significant surge (P < 0.01) in compression pressure immediately post-operation. This fact suggests that the internal pressure in the lower leg due to the potentially elastic stockings may increase while sensory deprivation due to spinal anesthesia is ongoing. This phenomenon may be attributable to complex factors, including altered fluid distribution subsequent to spinal anesthesia [6], transfusion to counteract hypotension, and autologous blood transfusion by uterine restoration. According to the practice guidelines for obstetric anesthesia [7], in CSs under spinal anesthesia, large infusions of colloidal or crystalloid fluid are administered to maintain uteroplacental blood flow as a way to counteract the relative reduction in circulating blood volume that occurs as a result of anesthesia-induced peripheral vasodilation. Such massive infusions prior to anesthesia are acceptable because massive hemorrhage may occur after the delivery of a child due to uterine relaxation. However, if uterine contractions are good and bleeding is minimal, a large amount of blood stored in the pregnant uterus is returned to the maternal circulatory system [8]. Uterine blood flow increases markedly during pregnancy, and in the case of a singleton pregnancy, the average uterine blood flow is 500-600 mL/min at 34-40 weeks [9]. In our secondary analysis in the present study, there was a difference in the immediate postoperative compressive pressure between patients with intraoperative blood loss of more than 1,000 g and those with intraoperative blood loss of less than 1,000 g. In other words, we speculate that when blood loss is relatively small, the return of blood stored in the gestational uterus, in addition to the massive infusion of fluid before anesthesia, maintains circulating blood volume, which in turn increases the compression pressure on the lower extremity. Given the small sample size in the secondary analysis and the absence of previous research on the relationship between the distribution of vascular beds and systemic blood volume throughout the body during spinal anesthesia, this phenomenon warrants additional descriptive studies. However, clinicians might be vigilant for potential occlusive neuropathy during persistent sensory nerve blockade in the lower extremities during the immediate postoperative period.

One distinguishing factor of a CS compared to other surgical procedures is that the mother must assume caregiving responsibilities for her child shortly after the operation. A previous study examining postpartum recovery after CS indicated that recovery is notably slower for several days following the surgery [10]. Moreover, pain, anxiety, and other physical abnormalities experienced after a CS can significantly impact the mother’s recovery and are linked to the development of postpartum depression [1]. It has been observed that if postpartum depression is left untreated, it can substantially affect not only the mother’s mental health but also the physical and mental development of the child [11]. Therefore, ensuring a healthy recovery with minimal complications after a CS is essential for positively influencing perinatal care. Although infrequent, the potential increase in intra-leg pressure from elastic stockings should be considered, as the development of strangulation neuropathy can interfere with both the physical and psychological recovery of pregnant women.

A limitation of this study is that none of the participants had postoperative peripheral neuropathy. Therefore, the clinical significance of the increased lower extremity compression pressure of POST observed in this study remains uncertain. However, the prevalence of peripheral nerve injury after delivery is small, approximately 0.3-2% of all deliveries [1], making studies with the power to detect differences in nerve injury unfeasible. Notably, four of the participants had pressure levels higher than those recommended for the elastic stockings used in this study; clearly, this increase may have impacted the increased risk of potential entrapment neuropathy. Therefore, measuring compression pressure immediately post-CS would be prudent, while alternative thromboprophylaxis should be opted for when an increase in compression pressure is detected. Furthermore, this is a small observational study and may not be generalizable to real populations. Further studies in a wider population are warranted.

## Conclusions

The application of elastic stockings could augment compression in the lower extremities immediately post-CS under spinal anesthesia, posing a risk of entrapment neuropathy. This study underscores the importance of balancing thromboembolic risk reduction with the potential risks of over-compression in patients who undergo CS with spinal anesthesia. Further research is needed to fully understand the clinical implications.
